# Electrical Capacitance Characteristics of Wood Chips at Low Frequency Ranges: A Cheap Tool for Quality Assessment

**DOI:** 10.3390/s21103494

**Published:** 2021-05-17

**Authors:** Jakub Lev, Václav Křepčík, Egidijus Šarauskis, František Kumhála

**Affiliations:** 1 Department of Physics, Faculty of Engineering, Czech University of Life Sciences Prague, Kamýcká 129, 165 00 Prague, Czech Republic; jlev@tf.czu.cz; 2 Department of Agricultural Machines, Faculty of Engineering, Czech University of Life Sciences Prague, Kamýcká 129, 165 00 Prague, Czech Republic; krepcik@tf.czu.cz; 3 Agriculture Academy, Institute of Agricultural Engineering and Safety, Vytautas Magnus University, Studentų g. 15a, LT-53362 Kaunas, Lithuania; egidijus.sarauskis@vdu.lt

**Keywords:** wood chips, moisture content, porosity, dielectric properties, low frequencies

## Abstract

Moisture content is one of the most important parameters related to the quality of wood chips that affects both the calorific and economic value of fuel chips. For industrial applications, moisture content needs to be detected quickly. For this purpose, various indirect moisture content measurement methods (e.g., capacitance, NIR, microwave, ECT, X-ray CT, and nuclear MR) have been investigated with different results in the past. Nevertheless, determining wood chip moisture content in real time is still a challenge. The main aim of this article was therefore to analyze the dielectric properties of wood chips at low frequencies (10 kHz–5 MHz) and to examine the possibility of using these properties to predict wood chip moisture content and porosity. A container-type probe was developed for this purpose. The electrical capacitance and dissipation factor of wood chips with different moisture content was measured by an LCR meter at 10 kHz, 50 kHz, 100 kHz, 500 kHz, 1 MHz, and 5 MHz frequencies. Wood chip porosity was also measured using a gas displacement method. Linear models for moisture content and porosity prediction were determined by backward stepwise linear regression. Mathematical model was developed to better understand the physical relationships between moisture content, porosity, and electrical capacitance. These models were able to predict the moisture content of observed quantities of wood chips with the required accuracy (R^2^ = 0.9–0.99). This finding opens another path to measuring the moisture content and porosity of wood chips in a relatively cheap and fast way and with adequate precision. In addition, principal component analysis showed that it is also possible to distinguish between individual wood chip fraction sizes from the information obtained.

## 1. Introduction

Moisture content (MC) is undoubtedly one of the most important parameters of the quality of wood chips, e.g., [1,2,3,4,5,6] and many others. It affects both the calorific and economic value of fuel chips. It is also well known that almost all of the physical and mechanical properties of wood are affected by its moisture content, e.g., [7,8,9] and others. Knowledge of wood or wood chip MC is therefore very useful. Although moisture is one of the basic natural characteristics of wood, its determination is a challenge, especially in real time, e.g., [10,11].

The direct, traditional, and still widely recognized method of determining the MC of wood chips is oven drying [2,4,5,6,7,9,12,13,14,15,16]. Although oven drying is a standard and standardized [4,7,16,17,18,19] method, many authors [2,4,5,6,12,13,15,16,17] also point out its disadvantages: the method is destructive, slow, and labor consuming, with potential sources of errors including mainly sampling errors or sample size, and it cannot be used for real-time measurements. Because of these disadvantages, the oven drying method is often unsuitable, for example, for measuring the properties of wood chips to be burned, etc.

Therefore, indirect wood chip MC measuring methods are being developed for online measurement. As early as in 2004, Nyström and Dahlquist [20] reviewed several methods of wood chip MC determination. They highlighted that biofuel is often heterogeneous and varies in MC from the extremes by 10–65%, but mostly between 45 and 55%. The dielectric and near infrared (NIR) principles of measurement were identified as the best of all the assessed methods. They also pointed out that principal component analysis (PCA) and partial least square (PLS) regression could be used for multivariate calibration of NIR.

In 2006, Jensen et al. [13] also compared MC determination in solid biofuels using dielectric and NIR methods. NIR research for wood and paper was then reviewed by Tsuchikawa [21], band assignments in NIR spectra of wood and wood components by Schwanninger et al. [22] and NIR spectroscopy suitable directly for monitoring moisture content and the density of solid wood was then reviewed Leblon et al. [23]. However, Trabelsi et al. [1] also highlighted some of the disadvantages of NIR technology. NIR-based methods measure surface moisture only and are affected by the size and geometry of the particles. This has also been confirmed by Dietsch et al. [7].

Another previously relatively widely researched indirect method of determining MC is based on microwave measurements. Nyström and Franzon [12] and recently Paz et al. [14] tested a radio frequency measurement system for biofuel MC determination. The tested radio frequency microwaves were within the range of 310 MHz to 1300 or 800 MHz, respectively. In 2010, Bogosanovic et al. [24] surveyed microwave noncontact wood measurement techniques, stating that the technology is mature, but more effort is needed to reach solutions applicable in practice. Paz et al. [25] used frequencies between 310 and 800 MHz for woody biomass moisture content above the fiber saturation point. Then, Trabelsi et al. [1] measured the bulk density and moisture content of peanut hull pellets with the microwave dielectric method, using frequencies between 5 GHz and 15 GHz. Daassi-Gnaba et al. [10] developed a system for measuring wood chip MC based on an antenna fully buried in a sample of wood chips. The authors used frequencies in the range of 50 MHz to 1.3 GHz. The conclusion was that the introduced MC prediction is suitable for real-time use. Recently, Daassi-Gnaba et al. [5] used microwaves (500 MHz–2 GHZ) to determine the MC of wood chips in a pile. The authors did not predict the exact MC value; they only recognized a MC category.

Parallel-plate capacitance sensors are also often used for MC determination. Kandala and Sundaram [26] measured the MC of grains and nuts (which is similar to wood chips) with this method at frequencies from 1 to 9 MHz. They measured the impedance and phase angle at two frequencies, 1 and 5 MHz, and also measured the capacitance value. They concluded that using a simple low-cost impedance meter and capacitance sensor, the MC of peanut and corn samples could be determined rapidly and non-destructively. Then, Kandala et al. [27] used a similar sensor for the measurement of the MC of different varieties of wheat. Impedance and phase angle were measured at 1 and 5 MHz. They concluded that the system was sufficiently accurate in the MC range between 9% and 25% and that measurements at two frequencies helped to eliminate measurement errors. Pan et al. [28] also tested coupled dielectric and impact sensor for simultaneous moisture content and mass flow of wood chips determination.

According to Dietsch et al. [7], among others, the following indirect methods to determine wood MC are used in practice: electrical resistance, dielectric (capacitive), microwave, radiometric and spectrometric. They noted that capacitive method is influenced by material density, temperature, and voltage frequency. The accuracy of MC determination of wood fuel chips using a handheld capacitance moisture meter was assessed by Fridh et al. [6]. Their conclusion was that the precision of this device was relatively low, especially on materials with a MC above 50%. For MC ranging from 20% to 50%, the error can be corrected using a calibration function.

Hultnäs and Fernandez-Cano [15] used dual-energy X-ray absorptiometry to determine the MC in wood chips. Lingren et al. [8] used dual-energy X-ray computed tomography (CT) to determine the MC in wood in three dimensions. They reported quite encouraging results. However, later Couceiro et al. [11] concluded that dual-energy X-ray CT technology cannot be used for the determination of MC in wood.

Another relatively widely studied measuring technology to be mentioned here is nuclear magnetic resonance (MR). Järvinen [16] stated that in principle, MR was a very accurate and reliable method of wood chip MC determination compared to other alternatives. On the other hand, the main challenge of using MR for online applications is the large installation and high investment costs. Fridh et al. [2] then used the same device as Järvinen, the Metso MR Moisture Analyzer. They concluded that the precision in determining MC and short measurement time makes this machine interesting for determining the MC of wood chips at customer sites. Xu et al. [9] demonstrated the possibility of MC profile measurement during wood drying by low-field MR using the Chinese-made device MesoMR23-060H-I. Aminti et al. [4] then used the Valmet MR (formerly Metso MR) device for woody biomass moisture content determination in southern European conditions. The device was relatively rapid.

Based on this literature review it can be stated that the MC of wood chips is an essential factor influencing both their calorific and economic value. For industrial applications, MC needs to be detected quickly. For this purpose, various indirect MC measurement methods have been investigated in the past, with varying results. Resistance and capacitance methods are often used in practice, but these have precision limits over a wider range of measurements. Devices capable of simultaneous measurement at different frequencies as well as container types, are advantageous. NIR-based technologies seem to be applicable but provide surface moisture readings only. Microwave technology is not the simplest but is usable and would probably benefit from further development. ECT can also be advantageous in some respects, but this relatively sophisticated method also requires further development to achieve the required accuracy. Another sophisticated method—X-ray CT—has failed in determining the MC of wood. Nuclear MR seems to be accurate and fast enough but is certainly not cheap. Advanced statistical (PCA, and PLS) and mathematical (modelling, machine learning) methods are often used for the purpose of indirect MC determination.

Based on these findings, the main aim of this article was therefore to analyze the dielectric properties of wood chips at low frequencies (10 kHz–5 MHz) and to examine the possibility of using these properties for the prediction of moisture content and wood chip porosity.

## 2. Materials and Methods

**Figure 1:**
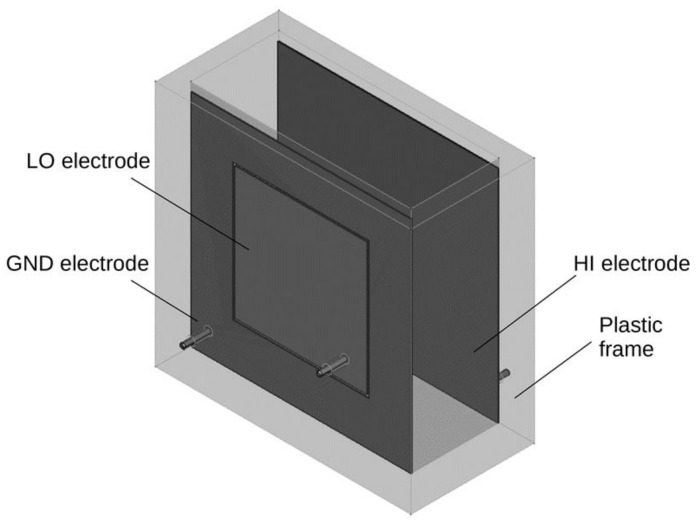
Depiction of the electrodes in the measuring box. HI electrode (high electric potential) sizes are 250 × 250 mm and LO electrode (low electric potential) sizes are 150 × 150 mm. The gap between GND electrode (grounded) and LO electrode is 2.5 mm. The electrodes are connected to terminals of an LCR bridge. The voltage between the terminals is 1 V (peak to peak).

A container-type probe was selected to use when measuring the dielectric properties (electrical capacitance and dissipation factor) of wood chips due to its advantages of higher accuracy and lower sensitivity to different fuels [13]. Therefore, a plastic box was constructed to be the probe. The box was made of 20 mm thick plastic plates that were glued and screwed together with plastic screws, ensuring the box was sufficiently strong. The inner dimensions of the box were 100 × 260 × 250 mm (resulting volume 6.5 dm^3^), and the outer dimensions were 140 × 280 × 290 mm. The arrangement of the electrodes is clear from Figure 1.

**Figure 2:**
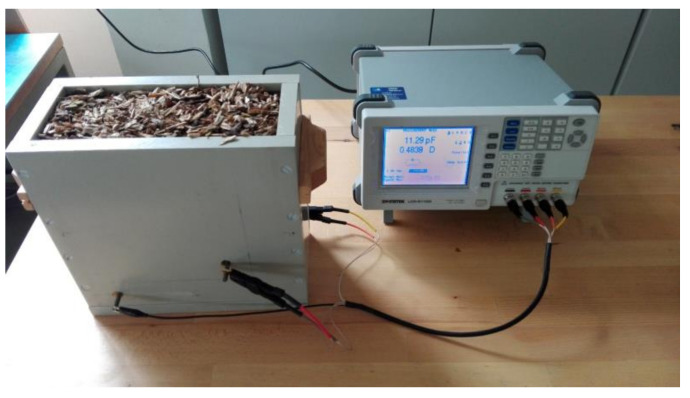
Connections between the measuring box and GW Intec 811G LCR meter for the measurement of the dielectric properties of wood chips.

Electrodes made of 1 mm stainless steel with the external dimensions of 250 × 250 mm were glued to the two larger inner walls of the box. The first electrode was divided into two centered parts. The outer part of the electrode had internal dimensions of 155 × 155 mm. The inner part of the electrode with the external dimensions of 150 × 150 mm was glued into this outer part and was insulated from the outer part by a 2.5 mm gap. From the second electrode as a whole and from both parts of the first electrode, electrical connections were routed through the plastic plates to connect to the measurement inputs of the measuring device. A GW Instek 811G LCR meter was used as the measuring device. The connections of the measuring apparatus for the measurement of the dielectric properties of wood chips is shown in Figure 2. In each of the individual measurements, the electrical capacitance and the dissipation factor were measured at frequencies of 10 kHz, 50 kHz, 100 kHz, 500 kHz, 1 MHz, and 5 MHz.

Prior to the start of the wood chip experiments, the assembly for measuring the dielectric properties of the wood chips was calibrated. An empty sensor (filled with air only) and a sensor completely filled with distilled water were calibrated.

Other important properties that characterize wood chips are bulk density and particle size distribution e.g., [1,13,29], whereas generally accepted method for wood chip particle size determination in wood industry is sieving analysis. Because dielectric properties are influenced by the presence of air [25], it was also decided to measure wood-chip porosity, although this measurement is quite complex. To measure it, an apparatus for measuring the volume of particulate solids was developed in our laboratory. This apparatus functions using the gas displacement method. The principle of this method is as follow. Measured sample is inserted in the device compartment of known volume, the appropriate inert gas is admitted, and then expanded into another precision internal volume. The pressures observed upon filling the sample chamber and then discharging it into a second empty chamber allow computation of the sample solid phase volume [30].

**Figure 3:**
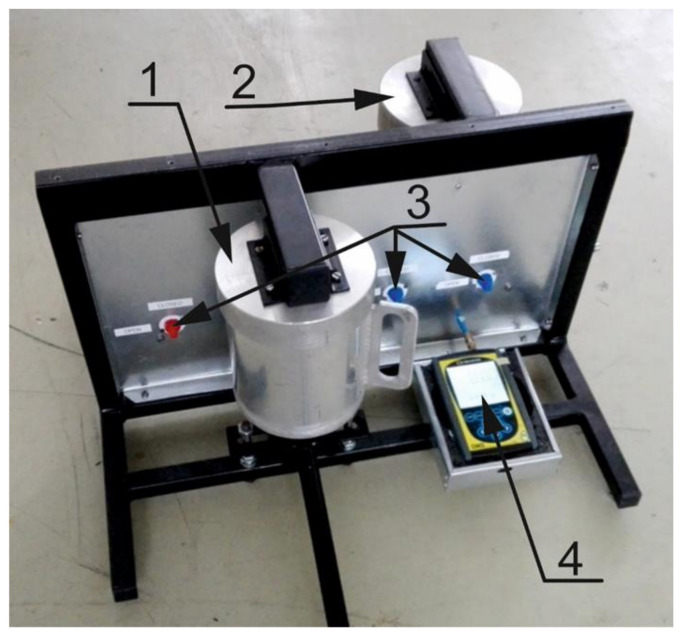
Assembly of the apparatus for measuring the volume of particulate solids (wood chip porosity). (**1**)—sample cylinder, (**2**)—reference cylinder, (**3**)—air valves, (**4**)—Cressto DMS 1 pressure gauge.

The basic components of this measuring device are a reference and sample cylinder. Both cylinders were welded from aluminum tubes with a wall-thickness of 10 mm and an aluminum sheet of the same thickness, to be strong enough so as not to change their volume. The measuring chambers of both cylinders have a diameter of 120 mm and a height of 150 mm, which represents a volume of about 1.7 dm^3^. The Cressto DMS 1 pressure gauge (Cressto Ltd., Rožnov pod Radhoštěm, Czech Republic) with a measuring range of ±2 kPa, measuring error 0.5%, was used to measure pressure differences in both chambers. The overall assembly of the volume measuring device is shown in Figure 3. Our previous experiments with this measuring instrument are described in more detail in [31]. Ambient air at a pressure of 1.5 kPa was used as a measuring gas due to its easy availability, although it is not an ideal gas for these purposes. Despite this fact, the resulting accuracy in determining the volume of a body of known dimensions (a steel cylinder with a diameter of 100 mm and a height of 140 mm) was 1.68% on average. When measuring wood chip volume using the gas displacement method, the internal pores of the material were also measured at lower material moisture contents.

**Figure 4:**
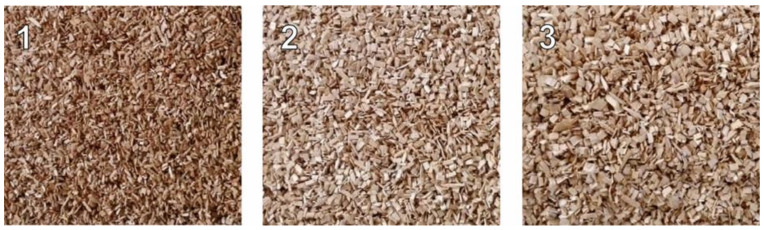
Individual fractions of pure oak wood chips after screen analysis. (**1**)—less than 3.15 mm, (**2**)—3.15 to 5 mm, (**3**)—greater than 5 mm.

The experiments were performed with pure and forest wood chips. Pure oak wood chips with individual particle sizes ranging from 1 to 10 mm were purchased from butcher’s supplies for measurement purposes. Pure wood chips were divided into 3 fractions with a volume of about 20 dm^3^ by screen analysis: (1) those less than 3.15 mm, (2) 3.15 to 5 mm, and (3) those greater than 5 mm. A view of the individual fractions of pure wood chips is shown in Figure 4. The intention was to capture the possible influence of fraction size on the measurement of dielectric properties. Initially, all 3 fractions thus obtained were placed in water at 12 °C for 24 h. This increased the moisture content of the pure wood chips to about 52% (wet basis). The material thus prepared was then placed in plastic bags, which were subsequently hermetically sealed.

A reference sample for moisture determination was taken from a measured fraction of the basic sample prior to measurement. The initial weight of the reference sample was about 100 g. A portion of the wood chips from the measured fraction was placed in a measuring box in order to measure its dielectric properties. The measured sample was poured into the measuring box using gravity only (it was not compressed).

This measurement was repeated 3 times for each fraction and frequency. The measuring box was emptied and filled with a new wood chips from the same fraction before each further measurement. The temperature of the wood chip fractions, and the air was measured and recorded at the beginning and at the end of the measurements. Care was taken to ensure that the temperature deviation between the beginning and end of the measurements was no more than ±1 °C and to keep the differences in ambient temperature in the laboratory to a minimum during all measurements (ambient temperature ranged from 20.6 to 22.3 °C).

After the dielectric measurements, the volume of each wood chip fraction was measured by gas displacement method using the apparatus for measuring the volume of particulate solids described above. As with the measurement of dielectric properties, the wood chips were poured into the measuring cylinder using gravity only. The measurement was performed 10 times for each fraction. After each individual measurement, the measuring cylinder was emptied and refilled with more chips from the same fraction.

After measuring the dielectric properties and volume of the wood chips at a particular MC, the entire fraction was partially dried in an oven at 105 °C together with the reference sample. During the drying process, the samples were mixed every 15 min to ensure uniform drying. The fraction was dried until the reference sample lost about 10 g of weight. After this reduction in moisture, the fraction (including the reference sample) was placed in a plastic bag for 24 h to homogenize its MC.

The dielectric properties and the volume of each wood chip fraction were then measured once more as described above. This procedure was repeated until the almost complete drying of the samples to dry matter was achieved. A total of 99 dielectric and 330 volume measurements were performed.

Then, the dry matter content of the reference sample was determined using oven-drying as a reference method. The oven drying method was carried out according to [18] (oven drying at 105 ± 2 °C in air atmosphere until constant mass was achieved).

Moisture content (wet basis) was calculated as follows:

(1) $$MC\left(\%\right)=\frac{weightbeforedrying-drymatterweight}{weightbeforedrying}\times 100,$$

From the dry weight of the reference sample thus obtained, it was possible to calculate material MC for each particular measurement. The MC of the individual fractions ranged from 0 to about 52% (wet basis).

Forest wood chips were obtained from imports delivered to Žatecká teplárenská Plc (a combined heat and power plant). These were always fresh wood chips containing a high proportion of moisture. Basic samples were collected in airtight plastic bags. The volume of basic samples was about 0.07 m^3^ (70 dm^3^). The samples were kept in their bags for 24 h to homogenize the moisture content. A total of three basic samples of wood chips with different composition were measured.

**Figure 5:**
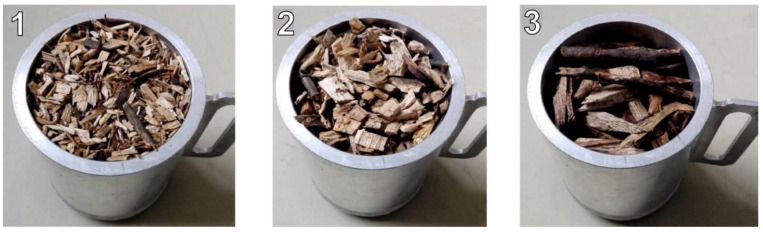
Individual fractions of forest wood chips after screen analysis, placed into the measuring cylinder for wood chip volume measurement. (**1**)—less than 10 mm, (**2**)—10 to 18 mm, (**3**)—larger than 18 mm.

At the beginning of each series of measurements, this basic sample was initially divided into 3 fractions with a volume of about 20 dm^3^ using screen analysis: (1) less than 10 mm, (2) 10 to 18 mm, and (3) greater than 18 mm. A view of the individual fractions of forest wood chips is shown in Figure 5.

The following steps in the measurement of the forest wood chips were the same as for the case of the pure wood chips. In this case, the MC of the individual fractions was slightly higher and ranged from 0 to about 58% (wet basis).

The measurement data were collected, and basic values were calculated using a spreadsheet (MS Excel). Next, statistical analyses (Pearson correlation and principal component analysis) were performed using the Python programming language (version 3.7, https://www.python.org, (accessed on 15 April 2021) together with supporting libraries (NumPy 1.17 https://numpy.org, (accessed on 15 April 2021), Scipy 1.3 https://www.scipy.org, (accessed on 15 April 2021), scikit-learn 0.21 https://scikit-learn.org, (accessed on 15 April 2021); [32]). Backward stepwise linear regression was performed using the R software (version 3.0.2, https://www.r-project.org, (accessed on 15 April 2021)).

## 3. Results and Discussion

Table 1 show the basic characteristics (% of MC and porosity) of the individual fractions of pure and forest wood chips at the monitored levels of MC. The achieved MC values are roughly within the usual range for wood chips (0–60%). The highest moisture content values were observed in both cases (pure and forest wood chips) for the first (the finest) fraction. This is probably associated with the fact that the first fraction contained the smallest particles, which were more effective at absorbing moisture.

Measured porosity was relatively high (in some cases over 80%). At this point, it should be noted that the method used for porosity measurement was based on gas displacement (as discussed before). When using this method, it is impossible to distinguish the inner pores inside the individual particles from the outer pores (voids) between them. The inner pores of the individual chips fill with water when moistened and the voids decrease with increasing MC. It is also clear that only open pores can be detected using the gas displacement method. It must therefore always be borne in mind that the porosity determined by measuring dielectric properties will be comparative to the porosity thus determined. The measured porosity values are larger for larger fractions (that is, the second and third), but the effect of MC on the measured porosity value is more significant than the effect of fraction size.

The standard deviation of the dielectric values ranged from 1 to 10% (in ca. 95% of cases). In several cases the standard deviation was higher, exceptionally up to 30%.

The aim of our research was mainly to investigate the possibility of predicting MC and porosity of wood chips. Therefore, in Table 2, we calculated the Pearson correlation coefficients of MC and pores with measured dielectric quantities (electrical capacitance and dissipation factor). In the case of measuring electrical capacitance it can generally be stated that at higher frequencies the resulting correlation values were higher. Exactly the opposite behavior was observed when measuring the dissipation factor. This behavior is related to phenomena whose occurrence is associated with the frequency of the applied electric field. In the case of electrical capacitance, this behavior can be caused by the Maxwell–Wagner polarization and electrode polarization effects. At higher frequencies, the polarization effects decrease, and so the measurement error may also decrease. The dissipation factor can be affected by ionic conductivity, as this generally decreases with increasing frequency [33].

**Figure 6:**
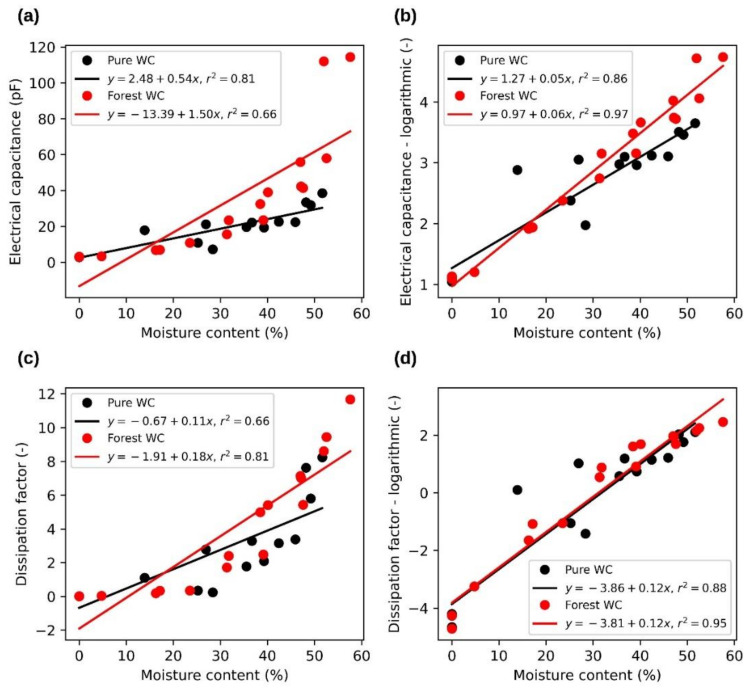
Relationships between wood chip moisture content and dielectric properties at 100 kHz. (**a**)—dependence of electrical capacitance on moisture content, (**b**)—dependence of logarithmic electrical capacitance on moisture content, (**c**)—dependence of dissipation factor on moisture content, (**d**)—dependence of logarithmic dissipation factor on moisture content.

**Figure 7:**
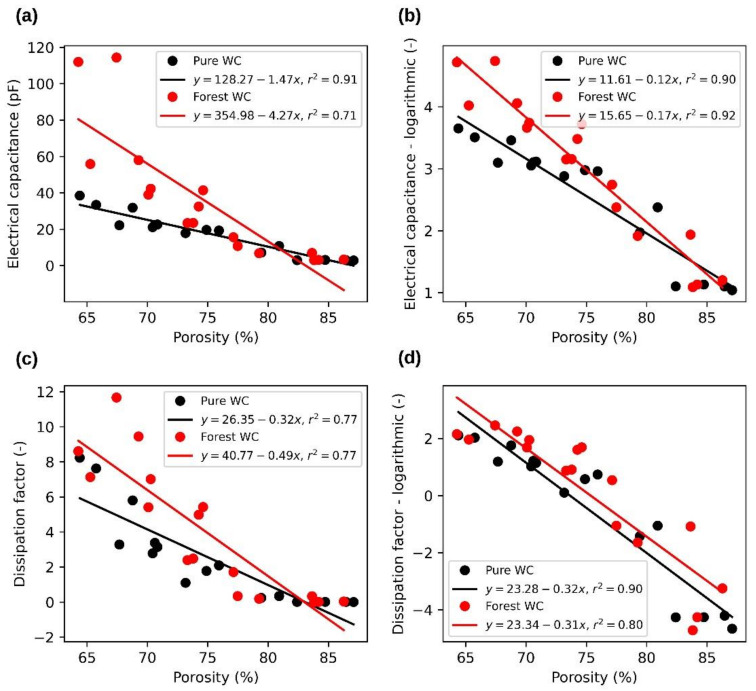
Relationships between wood chip porosity and dielectric properties at 100 kHz. (**a**)—dependence of electrical capacitance on porosity, (**b**)—dependence of logarithmic electrical capacitance on porosity, (**c**)—dependence of dissipation factor on porosity, (**d**)—dependence of logarithmic dissipation factor on porosity.

Typical examples of measured dependences of electrical capacity and loss factor on material MC and porosities are shown in Figure 6 and Figure 7. A frequency of 100 kHz was chosen for this example. Parts (a) and (c) of both figures show the measured data and parts (b) and (d) the data after logarithmization (the logarithmic value represents the natural logarithm of the original value.). The dependence curves of the monitored quantities measured at other observed frequencies were similar. In general, electrical capacitance and dissipation factor in dependence on MC increased faster at lower frequencies than at higher ones. Similarly, both observed values, depending on the porosity, decreased faster at lower frequencies than at higher frequencies.

Figure 6 shows the relationship between dielectric values and MC for both pure and forest wood chips. A partially improved linear dependence was achieved through logarithmization. This is especially evident in the case of electrical capacitance. The figures also distinguish between data for pure and forest wood chips. There is evidence of a greater variance in the measured values for forest wood chips, especially in the case of higher MC. This statement does not apply to low and zero MC, where in many cases the measured values are similar or even overlap. The probable cause of this phenomenon is the higher content of impurities in forest wood chips.

Figure 7 shows the relationship between dielectric values and porosity for both pure and forest wood chips. Unlike the previous measurement with MC, the electrical capacitance and dissipation factor decreases with increasing porosity. It can be seen that the effect of logarithmization on the linearization of the measured dependences was not as significant as in the case of MC. Even in this case, a greater variance was observed in the data for forest wood chips, but with smaller porosity values. As discussed earlier, the porosity is influenced primarily by the MC. Materials with a lower porosity, containing more moisture and impurities are, as in the previous case, probably the cause of greater variance in the measured values.

Linear models for MC and porosity prediction for both pure and forest wood chips are given in Table 3. All models were determined using backward stepwise linear regression in R software. Models were calculated for both measured and logarithmic data. Multiple and adjusted coefficients of determination values were calculated for all models. In the case of forest wood chips, logarithmization contributed to a significant simplification of the models, but at the cost of reducing the coefficients of determination.

However, linear models were not simplified by logarithmization in the case of pure wood chips. In this case, after logarithmization of the measured data, more complex linear models resulted, but with a better coefficient of determination. MC was more accurately predicted by these linear models for forest wood chips, while porosity was more accurately predicted for pure wood chips.

**Figure 8:**
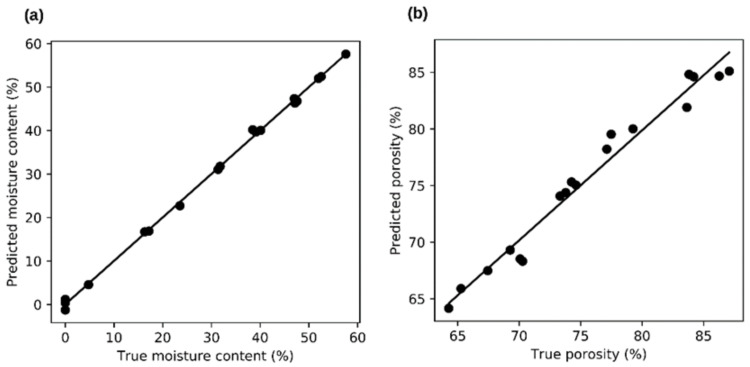
Examples of relationships between real data and predicted data. Plotted data for forest wood chips without logarithmic adjustment. (**a**)—linear model for moisture content, R^2^ = 0.997. (**b**)—linear model for porosity, R^2^ = 0.962.

Figure 8 plots the relationships between measured and predicted values for the MC and porosity of forest wood chips. It is clear from this figure that using the proposed models, it is possible to predict the MC of wood chips and their porosity with adequate accuracy, even when measuring their electrical properties at low frequencies. The adjusted coefficient of determination for MC prediction achieved the value R^2^ = 0.997 and for porosity prediction it was R^2^ = 0.962. In both cases, this is quite an encouraging result.

Kandala et al. [27] reported coefficient of determination R^2^ = 0.99 in the case of wheat moisture measurements by a parallel-plate capacitive sensor at two frequencies—1 and 5 MHz—in the MC measurement range from 9 to 25%. Our result of predicting the MC of forest wood chips is fully comparable with those results, with the fact that our measurements ranged from a MC of 0 to almost 58%. In terms of practical use, our measuring device was very similar in comparison to the one used by these authors. Based on our results presented in this paper and this comparison, it can be stated that measuring the electrical properties of wood chips at lower frequencies is undoubtedly a promising method to predict their MC fast and with adequate precision.

In the case of using ECT technology, the best reported coefficient of determination R^2^ = 0.93 can be achieved in the prediction of MC when using an NIR sensor R^2^ = 0.9 [3]. However, the advantage of these methods is that both can be continuous.

When assessing the accuracy of the handheld electric capacitance moisture meter, [6] reported relatively low precision (±3.8%). The authors also pointed out that this device should not be used on materials with a MC above 50%. In the range from 20% to 50%, a calibration function can be used to correct the systematic underestimation of MC. However, the measuring procedure is simpler compared to container-type probes. The assessed Wile Bio Moisture meter uses a dish probe with a tip as its MC sensor.

Aminti et al. [4] tested a magnetic resonance moisture meter for wood chip MC determination. This device was able to predict wood chip MC with R^2^ = 0.96. This type of moisture meter uses a container-type probe. Since the coefficient of determination achieved by us was slightly better and that we used a very similar type of probe, it can be stated that both devices are fully comparable in terms of function. However, the advantage of our solution is that measuring the electrical properties of wood chips at low frequencies is in principle significantly easier than magnetic resonance, and therefore, as a result, our device should be significantly cheaper [27]. Another advantage of our device is that we can determine not only MC, but also porosity with good accuracy.

From the point of view of possible practical uses of the results of our measurements, simplified linear models obtained after logarithmization of the measured data are interesting. In our case, it can be seen in Table 3, that when predicting the MC of forest wood chips with an adjusted R^2^ = 0.985, it is enough to measure electrical capacity at 1 MHz and dissipation factor at 500 kHz frequencies. Moreover, it is also possible to predict porosity of forest wood chips with a coefficient of determination R^2^ = 0.933 from the values at an electrical capacity of 1 MHz. In other words, to be able to predict MC and porosity with adequate precision, it was sufficient to measure the electrical properties of forest wood chips at only two frequencies, 1 MHz and 500 kHz.

A mathematical model was developed to better understand the physical relationships between moisture content, porosity, and electrical capacitance.

The following equation can be used to describe the dependence of electrical capacity on moisture content and porosity [34]:

*ε*^1/*N*^ = *v*_1_ *ε*_1_^1/*N*^ + *v*_2_ *ε*_2_^1/*N*^, (2)

where *ε* is the effective relative permittivity of the mixture, *ε*_1_ and *ε*_2_ are the values of the relative permittivity of the individual components and *v*_1_ and *v*_2_ are the respective volume fractions, where the sum of both fractions must be equal to one. *N* represents an empirical parameter that considers the geometric properties of the particles. In this case it is considering that *ε*_1_ and *v*_1_ represent a solid part of the wet chips and *ε*_2_ and *v*_2_ represent air. If the relative permittivity of air is considering equal to one and *v*_2_ is replacing by the porosity *P*, the effective permittivity of the wood chips can be defined as follows:

*ε = [(*1 − *P) ε*_1_^1/*N*^*+ P]^N^*. (3)

The value of *ε*_1_ is primarily influenced by the wood chips moisture content. The second order polynomial function was chosen as the best compromise of the dependencies we tested for the relationship between *ε*_1_ and *MC*:

*ε*_1_ = *k*_0_ + *k*_1_*MC + k*_2_*MC*^2^, (4)

where *k*_0_, *k*_1_, and *k*_2_ are the coefficients of the polynomial. Substitute Equation (4) into Equation (3) and modify a mathematical expression. A final equation for the electrical capacity in the measuring capacitor *C* can be obtained by this way:

*C = ε*_0_*S/d [(*1 − *P) (k*_0_ + *k*_1_*MC* + *k*_2_*MC*^2^)^1/*N*^ + *P ]^N^*, (5)

where *ε*_0_ is the permittivity of the vacuum (*ε*_0_ = 8.85E − 12 F m^−1^), *S* is the electrode area of the measuring capacitor and *d* is the distance between the electrodes.

The capacitance data obtained at frequencies 100 kHz, 500 KHz, 1 MHz, and 5 MHz for both forest and pure wood chips were than fitted by Equation (5). The data obtained at lower frequencies was not used because an effect of electrode polarization is assumed. Determined equation parameters and coefficients of determination (*r*^2^) are in the Table 4. The equation coefficients were determined using the ‘optimize.curve_fit’ function which is available in Scipy library. This tool allows use bounds for sought coefficients which were used for coefficients *k*_0_ and *N*. The coefficient *k*_0_ could represent a relative permittivity of a dry wood and therefore it is assumed that its value should be higher to one. The value of the coefficient *N* is expected around 2 or 3 [25,34,35]; therefore, this coefficient was sought in the range from 0 to 10.

The coefficient of determination in the Table 4 shows relatively good consistency of the model with the data. The values range around 0.96 for 500 kHz and around 0.97 for 1 MHz frequency. The values of the equation coefficients are similar for forest and pure wood chips. It can be stated that the calculated coefficients of the equations are more influenced by the measuring frequencies than by the type of wood chips. The determined coefficient *N* range from 1.23 to 3.06. This agrees with previous works [25,35]. However, the change in the coefficient together with the frequency indicates a connection of this coefficient with the change in the effect of some types of polarization (Maxwell–Wagner phenomena).

**Figure 9:**
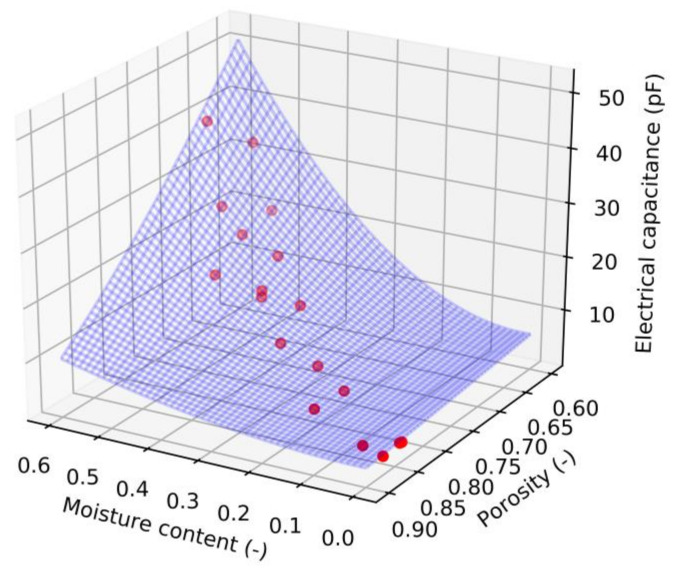
Plotted measured capacitance data (red points) for forest wood chips at1 MHz frequency and mathematical model based on the Equation (5)—blue surface.

An example of the plotted data (forest wood chips and frequency 1 MHz) and model is shown in the Figure 9. This graph shows the expected behavior, namely that the electrical capacity increases with increasing moisture content and, conversely, decreases with increasing porosity. The graph also shows that the porosity correlates with moisture content and the porosity variance for a specific moisture content is limited.

**Figure 10:**
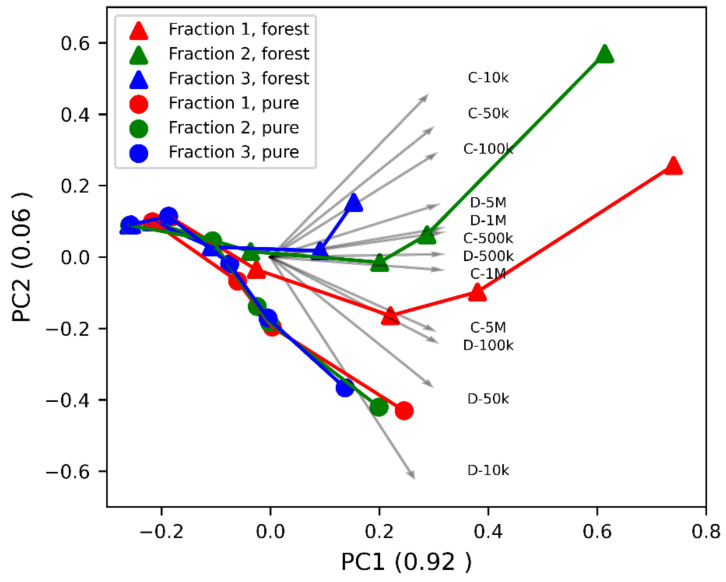
Principal component analysis (PCA) of the dielectric data for pure and forest wood chips. The arrows show a contribution of the dielectric quantities to individual components. The values of the principal components are normalized and range from −1 to 1.

As it can be seen from the previous text, there were correlations between the individual variables in the extensive data set we measured. In order to better understand the information that the all the measured data represent (including low frequencies and dissipation factor), we decided to use a PCA method. A graphical presentation of the results of this method is shown in Figure 10.

PCA in Figure 10 closely described the variability for the measured dielectric data. As is clear from this figure, the vast majority of variability is explained by the first principal component (92%), the second already adds only 6%. Together, these two principal components explain 98% of the variability and the other components are therefore no longer relevant.

The individual wood chip samples form curves (distinguished by different colors and dots in the picture), which, however, start from a single point. This point corresponds to a dry wood chip. As the MC of the wood chips increases, several curves (chip samples) can be distinguished. This is particularly apparent for forest wood chips, where the individual fractions can be safely distinguished from one other. Pure wood chips can be distinguished as a whole. However, their individual fractions cannot be distinguished from one another.

When assessing the contributions of individual dielectric quantities to the first and second principal component, it is clear that both the capacitance and the dissipation factor are arranged according to the frequencies at which they were measured. The second principal component, thanks to which some fractions can be distinguished, consists mainly of data measured at lower frequencies (10 kHz, 50 kHz, and 100 kHz). This means that the second principal component represent effects such as ionic conductivity, Maxwell—Wagner polarization and electrode polarization. The last effect probably plays an important role in this case. Electrode polarization is a process that takes place on the contact surfaces of the material and the electrodes and does not represent the true dielectric properties of the material [36]. However, the relationship between electrode polarization and the contact surface (smaller particles have better contact with the electrode) can explain the ability to differentiate between certain fractions. It follows from this fact that measuring dielectric quantities at lower frequencies also has the potential to explain other wood chips properties, such as their size composition.

## 4. Conclusions

It was found that dielectric properties obtained at low frequencies (10 kHz–5 MHz) from parallel plate capacitor inserted in container-type probe can be used for the sufficiently accurate prediction of wood chip moisture content and porosity. The obtained coefficients of determination ranged from 0.9 to 0.99. Only in exceptional cases, a higher variance of the measured dielectric properties was recorded when measuring wood chips with high moisture content at low frequencies. This finding opens another path to measure the moisture content and porosity of wood chips relatively cheaply, fast, and with adequate precision.

The measuring frequencies affected the results of the mathematical model of the dependence of electrical capacity, porosity, and moisture content more than the type of wood chips. Principal component analysis showed that individual fractions of forest wood chips are also distinguishable. The differences between the fractions are especially noticeable with higher moisture content and these differences are mainly associated with data that were measured at lower frequencies. It can be assumed that this observation is related to the different size composition in the individual wood chip fractions.

## Figures and Tables

**Table 1:** Moisture content (MC) and porosity (P) of pure and forest wood chips.

Pure Wood Chips						
MC level	Fraction 1		Fraction 2		Fraction 3	
	MC (%)	P (%)	MC (%)	P (%)	MC (%)	P (%)
0.	0	82.4 ± 0.2	0	86.4 ± 0.5	0	84.7 ± 0.9
1.	28.4	79.4 ± 0.5	13.9	73.2 ± 0.8	25.2	80.9 ± 0.5
2.	39.3	75.9 ± 1.4	26.9	70.4 ± 0.4	35.5	74.9 ± 1.0
3.	45.9	70.9 ± 0.6	36.7	67.7 ± 0.7	42.4	70.8 ± 0.5
4.	51.7	64.4 ± 0.2	48.2	65.8 ± 1.4	49.2	68.8 ± 1.2
**Forest Wood Chips**						
**MC level**	**Fraction 1**		**Fraction 2**		**Fraction 3**	
	**MC (%)**	**P (%)**	**MC (%)**	**P (%)**	**MC (%)**	**P (%)**
0.	0	84.2 ± 0.1	0	83.8 ± 1.4	0	87.1 ± 0.2
1.	23.5	77.5 ± 0.7	16.3	79.3 ± 0.5	4.8	86.3 ± 0.2
2.	39.1	73.8 ± 0.5	31.8	73.3 ± 0.9	17.2	83.6 ± 0.6
3.	47.1	70.3 ± 1.0	40.1	70.1 ± 0.4	31.4	77.1 ± 1.1
4.	52.5	69.3 ± 1.8	47.0	65.3 ± 1.3	38.5	74.3 ± 2.6
5.	57.6	67.4 ± 0.8	52.0	64.3 ± 0.3	47.6	74.6 ± 1.9

**Table 2:** Pearson correlation coefficients between measured electrical capacity (C), dissipation factor (D), moisture content (MC) and porosity (P) at different frequencies.

	C_10k_	D_10k_	C_50k_	D_50k_	C_100k_	D_100k_	C_500k_	D_500k_	C_1M_	D_1M_	C_5M_	D_5M_
MC	0.66	0.86	0.70	0.87	0.74	0.85	0.83	0.81	0.87	0.80	0.90	0.78
P	−0.62	−0.90	−0.69	−0.86	−0.73	−0.84	−0.83	−0.79	−0.87	−0.78	−0.91	−0.77

**Table 3:** Linear models for moisture content (MC) and porosity (P) determined by backward stepwise linear regression. C—electrical capacitance, D—dissipation factor, WC—wood chips.

Variant	Model	Adjusted R^2^	Multiple R^2^
MC forest WC	*MC* = −0.137 + 0.018 *C*_10*k*_ − 0.312 *C*_50*k*_ + 0.640 *C*_100*k*_ − 1.464 *C*_500*k*_ + 1.321 *C*_1*M*_ −0.162 *C*_5*M*_ − 0.022 *D*_10*k*_ + 0.223 *D*_50*k*_ − 0.397 *D*_100*k*_ + 1.478 *D*_5*M*_	0.997	0.999
Porosity forest WC	*P = 0.92 + 0.003 C_10k_* − *0.023 C_50k_ + 0.174 C_500k_* − *0.18 C_1M_*	0.962	0.971
MC pure WC	*MC* = 0.1 + 0.179 *C*_10*k*_ − 0.51 *C*_100*k*_ + 0.306 *C*_500*k*_ + 0.592 *D*_10*k*_ − 5.333 *D*_50*k*_ + 5.659 *D*_100*k*_	0.905	0.946
Porosity pure WC	*P* = 0.991 + 0.008 *C*_10*k*_ − 0.053 *C*_5*M*_ + 0.013 *D*_10*k*_ − 0.875 *D*_500*k*_ + 0.978 *D*_1*M*_	0.976	0.985
MC forest WC Log	*MC* = 0.018 + 0.131 *C*_1*M*_ + 0.037 *D*_500*k*_	0.985	0.987
Porosity forest WC Log	*P* = 0.94 − 0.077 *C*_1*M*_	0.933	0.937
MC pure WC Log	*MC* = −0.627 + 1.439 *C*_10*k*_ − 3.405 *C*_100*k*_ + 6.454 *C*_500*k*_ −6.087 *C*._5*M*_ + 0.739 *D*_10*k*_ − 0.431 *D*_50*k*_ − 0.35 *D*_500*k*_ + 1.702 *D*_1*M*_ − 2.54 *D*_5*M*_	0.978	0.992

**Table 4:** Calculated coefficients of the mathematical model based on the Equation (5) and coefficients of determination.

Variant	*r* ^2^	*k*_0_	*k_1_*	*k*_2_	*N*
forest 100 kHz	0.909	110.11	−1322.83	4334.03	2.96
forest 500 kHz	0.968	11.93	−85.05	583.26	1.87
forest 1 MHz	0.976	7.06	−8.42	233.98	1.44
forest 5 MHz	0.914	3.94	41.29	27.96	1.24
pure 100 kHz	0.901	143.73	−1612.98	4909.44	3.06
pure 500 kHz	0.960	13.07	−95.74	600.71	1.87
pure 1 MHz	0.969	7.51	−12.16	239.45	1.43
pure 5 MHz	0.886	3.97	41.72	26.88	1.23

## References

[1] Trabelsi S., Paz A.M., Nelson S.O. (2013). Microwave dielectric method for the rapid, nondestructive determination of bulk density and moisture content of peanut hull pellets. Biosyst. Eng..

[2] Fridh L., Volpé S., Eliasson L. (2014). An accurate and fast method for moisture content determination. Int. J. For. Eng..

[3] Pan P., McDonald T.P., Via B.K., Fulton J.P., Hung J.Y. (2016). Predicting moisture content of chipped pine samples with a multi-electrode capacitance sensor. Biosyst. Eng..

[4] Aminti G., Cinotti A., Lombardinia C., Spinellia R., Picchia G. (2018). Industrial stress-test of a magnetic resonance moisture meter for woody biomass in southern European conditions. Fuel Process. Technol..

[5] Gnaba H.D., Oussar Y., Merlan M., Ditchi T., Géron E., Holé S. (2018). Moisture content recognition for wood chips in pile using supervised classification. Wood Sci. Technol..

[6] Fridh L., Eliasson L., Bergström D. (2018). Precision and accuracy in moisture content determination of wood fuel chips using a handheld electric capacitance moisture meter. Silva. Fenn..

[7] Dietsch P., Franke S., Franke B., Gamper A., Winter S. (2015). Methods to determine wood moisture content and their applicability in monitoring concepts. J. Civil. Struct. Health Monit..

[8] Lindgren O., Seifert T., Du Plessis A. (2016). Moisture content measurements in wood using dual-energy CT scanning—A feasibility study. Wood Mater. Sci. Eng..

[9] Xu K., Lu J., Gaoa Y., Wu Y., Li X. (2017). Determination of moisture content and moisture content profiles in wood during drying by low-field nuclear magnetic resonance. Dry Technol..

[10] Daassi-Gnaba H., Oussar Y., Merlan M., Ditchi T., Géron E., Holé S. (2017). Wood moisture content prediction using feature selection techniques and a kernel method. Neurocomputing.

[11] Couceiro J., Lindgren O., Hansson L., Söderström O., Sandberg D. (2019). Real-time wood moisture-content determination using dual-energy X-ray computed tomography scanning. Wood Mater. Sci. Eng..

[12] Nyström J., Franzon B. Radio Frequency System for Measuring Characteristics of Biofuels. Proceedings of the IEEE Conference IMTC 2005—Instrumentation and Measurement Technology Conference.

[13] Jensen P.D., Hartmann H., Böhm T., Temmerman M., Rabier F., Merete M. (2006). Moisture content determination in solid biofuels by dielectric and NIR reflection methods. Biomass. Bioenerg..

[14] Paz A., Nyström J., Thorin E. Influence of Temperature in Radio Frequency Measurements of Moisture Content in Biofuel. Proceedings of the IEEE Conference IMTC 2006—Instrumentation and Measurement Technology Conference.

[15] Hultnäs M., Fernandez-Cano V. (2012). Determination of the moisture content in wood chips of Scots pine and Norway spruce using Mantex Desktop Scanner based on dual energy X-ray absorptiometry. J. Wood Sci..

[16] Järvinen T. (2013). Rapid and Accurate Biofuel Moisture Content Gauging Using Magnetic Resonance Measurement Technology.

[17] Österberg P., Heinonen M., Ojanen-Saloranta M., Mäkynen A. (2016). Comparison of the performance of a microwave-based and an NMR-based biomaterial moisture measurement instrument. Acta IMEKO.

[18] EN 14774-1:2009 Solid Biofuels—Determination of Moisture Content—Oven Dry Method—Part 1: Total Moisture—Reference Method. https://www.sis.se/api/document/preview/71672/.

[19] ISO 18134-1:2015 Solid Biofuels—Determination of Moisture Content—Oven Dry Method—Part 1: Total Moisture—Reference Method. https://www.iso.org/standard/61538.html.

[20] Nyström J., Dahlquist E. (2004). Methods for determination of moisture content in woodchips for power plants—A review. Fuel.

[21] Tsuchikawa S. (2007). A Review of Recent Near Infrared Research for Wood and Paper. Appl. Spectrosc. Rev..

[22] Schwanninger M., Rodrigues J.C., Fackler K. (2011). A review of band assignments in near infrared spectra of wood and wood components. J. Near Infrared Spectrosc..

[23] Leblon B., Adedipe O., Hans G., Haddadi A., Tsuchikawa S., Burger J., Stirling R., Pirouz Z., Groves K., Nader J. (2013). A review of near-infrared spectroscopy for monitoring moisture content and density of solid wood. For. Chron..

[24] Bogosanovic M., Al Anbuky A., Emms G.W. (2010). Overview and comparison of microwave noncontact wood measurement techniques. J. Wood Sci..

[25] Paz A., Thorin E., Topp C. (2011). Dielectric mixing models for water content determination in woody biomass. Wood Sci. Technol..

[26] Kandala C.V.K., Sundaram J. (2010). Nondestructive Measurement of Moisture Content Using a Parallel-Plate Capacitance Sensor for Grain and Nuts. IEEE Sens. J..

[27] Kandala C.V.K., Sundaram J., Puppala N., Settaluri V. (2012). Nondestructive measurement of moisture content of different varieties of wheat using a single calibration with a parallel-plate capacitance sensor. Trans. ASABE.

[28] Pan P., McDonald T., Fulton J., Via B., Hung J. (2017). Simultaneous Moisture Content and Mass Flow Measurements in Wood Chip Flows Using Coupled Dielectric and Impact Sensors. Sensors.

[29] Kofman P.D. (2006). Quality Wood Chip Fuel. http://woodenergy.ie/media/coford/content/publications/projectreports/cofordconnects/finalfuelquality.pdf.

[30] Serpil S., Servet G.S. (2006). Physical Properties of Foods.

[31] Křepčík V., Lev J., Kumhála F. (2017). Development and testing of apparatus for wooden chips voids measurement. Agron. Res..

[32] Pedregosa F., Varoquaux G., Gramfort A., Michel V., Thirion B., Grisel O., Blondel M., Prettenhofer P., Weiss R., Dubourg V. (2011). Scikit-learn: Machine Learning in Python. J. Mach. Learn. Res..

[33] Skierucha W., Wilczek A., Szyplowska A. (2012). Dielectric spectroscopy in agrophysics. Int. Agrophys..

[34] Ferre P., Topp G., Baltes H., Göpel W., Hesse J. (2000). Time-domain reflectometry techniques for soil water content and electrical conductivity measurements. Sensors Update.

[35] Nelson S. (2015). Dielectric Properties of Agricultural Materials and Their Applications.

[36] Oh M., Kim Y., Park J. (2007). Factors affecting the complex permittivity spectrum of soil at a low frequency range of 1 kHz–10 MHz. Environ. Geol..

